# Effect of eye rubbing on corneal biomechanical properties in myopia and emmetropia

**DOI:** 10.3389/fbioe.2023.1168503

**Published:** 2023-06-06

**Authors:** Xia Li, Anji Wei, Yujing Yang, Jiaxu Hong, Jianjiang Xu

**Affiliations:** ^1^ Department of Ophthalmology, Shanghai Aier Eye Hospital, Shanghai, China; ^2^ Department of Ophthalmology, Eye, Ear, Nose, and Throat Hospital, Shanghai Medical College, Fudan University, Shanghai, China; ^3^ Department of Ophthalmology, The Affiliated Hospital of Guizhou Medical University, Guiyang, China

**Keywords:** eye rubbing, corneal biomechanical properties, SP-A1, susceptible, Corvis ST

## Abstract

**Purpose:** To investigate short-term changes in corneal biomechanical properties caused by eye rubbing in myopia and emmetropia and compare the different responses between the two groups.

**Methods:** This was a prospective observational study of 57 eyes of 57 healthy subjects aged 45 years and younger. The participants were divided into myopia and emmetropia groups. All the subjects underwent eye rubbing by the same investigator using the same technique. Biomechanical parameters were recorded using the Corvis ST device before and after 1 min of eye rubbing. One week later, all the participants underwent the test again. Statistical methods were employed to compare the differences between the data from before and after the 1 min of eye rubbing and demonstrate the different responses of the two groups.

**Results:** After 1 min of eye rubbing, smaller SP-A1 (*p* < 0.001), higher deformation and deflection amplitudes (*p* < 0.001, *p* = 0.012), higher peak distances (*p* < 0.001), earlier A1 times (*p* < 0.001), faster velocities (*p* < 0.001), and lower maximum inverse radii (*p* = 0.004) were observed. According to the automatic linear modeling analysis, the refractive states (B = −5.236, *p* = 0.010) and biomechanically corrected intraocular pressure (bIOP) (B = 0.196, *p* = 0.016) had influenced a decrease in the stiffness parameter at the first applanation (SP-A1). The central corneal thickness (CCT) had decreased only in the myopia group (*p* = 0.039). The change of SP-A1 in amplitude was larger in the myopia group than in the emmetropia group (*p* < 0.001). All the parameters returned to the baseline level 1 week later.

**Conclusion:** Eye rubbing appears to alter corneal biomechanical properties temporarily and make the cornea softer, especially for myopic young patients.

## 1 Introduction

Eye rubbing is a common physiological response to discomfort, fatigue, or ocular itch. It is also a natural behavior before or after sleep (Greiner et al., 1997; McMonnies and Boneham, 2003). However, when eye rubbing is performed vigorously or the duration of rubbing episodes are extended, it is potentially harmful, especially to the cornea, which is directly exposed to the rubbing action by virtue of its location (McMonnies and Boneham, 2003; Ahuja et al., 2020; Rabinowitz et al., 2021; Sahebjada et al., 2021; Santodomingo-Rubido et al., 2022).

Biomechanical corneal properties depend on the composition and internal structure of the cornea. To date, two devices based on non-contact tonometry have been developed to describe corneal biomechanics *in vivo*: the ocular response analyzer (ORA) (Reichert, Inc., Depew, NY) (Luce, 2005; Chong and Dupps, 2021) and the Corvis ST tonometer (OCULUS Optikgeräte GmbH, Wetzlar, Germany) (Ambrósio Jr et al., 2013). The Corvis ST captures corneal dynamic deformation under air pulse excitation with the aid of corneal visualization using the Scheimpflug technology that is used to evaluate ocular biomechanics (Roberts et al., 2017). Furthermore, by using the Vinciguerra Screening Report and Corvis Biomechanical Index to describe corneal mechanical properties, confounding factors of intraocular pressure and central corneal thickness that affect the dynamic corneal response parameter are decreased.

Among all the parameters achieved by the Corvis ST, the stiffness parameter at the first applanation (SP-A1) represents the stiffness of the cornea (Roberts et al., 2016). It is defined as the difference between the air puff pressure at the corneal surface and the biomechanically corrected intraocular pressure at the first applanation divided by the deflection amplitude (Roberts et al., 2016). Also, it has been proven to be a clinically useful parameter to demonstrate corneal stiffness values (Roberts et al., 2016). Many previous studies have demonstrated that, in some cases, SP-A1 significantly decreases when the cornea becomes softer or thinner (Luce, 2005; Dawson et al., 2008; Chong and Dupps, 2021).

The cornea is composed of lamellar layers that are resistant to longitudinal stress but vulnerable to shear and radial stress (Dawson et al., 2008). Eye rubbing, especially cyclical rubbing and the associated mechanical load may interfere with the interlamellar bonds (Dawson et al., 2008). Flexure of the fibrils and relaxation of the lamellae in an indented area of the cornea may occur in response to rubbing, whereas a spike in intraocular pressure may induce stress of corneal lamellae in areas adjacent to the indented area. Eye rubbing results in potential micro trauma at the cellular level (McMonnies, 2009), leading to cytokine release, myofibroblast differentiation, and even shape changes of the corneal tissue (McMonnies, 2009; Chervenkoff et al., 2017; Scotto et al., 2021).

The changes caused by eye rubbing could result in changes in corneal biomechanical properties. Previous studies, by using the ORA, have shown that eye rubbing decreases corneal hysteresis, the corneal resistance factor (Liu et al., 2011). The Corvis ST can display real-time images of the cornea in response to the air puff pressure and provide accurate and reliable corneal biomechanical parameters, and this has been widely used in clinics. However, there has been no previous research done on corneal biomechanical property changes after eye rubbing using the Corvis ST.

The purpose of the present study is to investigate the influence of eye rubbing on corneal biomechanics *in vivo* using the Corvis ST device and compare the different responses between the myopia and emmetropia groups.

## 2 Materials and methods

This study followed the tenets of the Declaration of Helsinki and was approved by the ethics committee of the Eye, Ear, Nose, and Throat Hospital, Shanghai, China (2015044-1). Written informed consent was obtained from all the subjects, which included consent to allow the use of their clinical data for scientific purposes. The same experienced investigator conducted all the eye rubbing tests.

### 2.1 Patients

This comparative study included 57 eyes of 57 subjects aged 45 years and younger. Subjects with keratoconus were not included in the study for ethical reasons. Contact lens wearers were also excluded. Other exclusion criteria included subjects who had ocular or systemic allergy conditions, had ocular surgery, or had used topical or systemic medications. All the participants underwent a thorough ophthalmologic examination and optometry. Emmetropia (Jiang et al., 2020) was defined as uncorrected visual acuity (UCVA) ≥5.0 and spherical equivalent refraction (SER) +0.50 ≥ SER ≥ −0.50 D. Twenty-eight subjects of the study population had myopia, while twenty-nine had emmetropia.

### 2.2 Eye rubbing technique

Eye rubbing was carried out by the same investigator. The participants were instructed to maintain a steady central gaze position, with the contralateral eye opened during the procedure to ensure exposure of the central corneal area to rubbing. A circular eye rubbing movement over the eyelids was used, applying a constant pressure toward the center of the cornea. The rubbing massage lasted for exactly 1 min, without interruption. The amount of force applied during rubbing was limited, so as to not cause undue damage to the eye. The left hand was used to rub the right eye and the right hand was used to rub the left eye using the knuckle of the index finger. The measurement was carried out immediately after eye rubbing. We chose the right eye for analysis to avoid correlation interference.

### 2.3 Measurement of corneal biomechanical parameters

The Corvis ST (software version 1.3r1469) uses a precisely metered air pulse to deform the cornea, along with an ultrahigh speed camera, which utilizes the Scheimpflug geometry to capture images of the horizontal meridian at greater than 4,300 frames per second, resulting in 140 images during a 30-ms air puff (Ambrósio Jr et al., 2013) The output parameters of the Corvis ST include intraocular pressure (IOP), biomechanically corrected intraocular pressure (bIOP), central corneal thickness (CCT), time from the initiation of the air puff until the first and second applanation (A1 T and A2 T), length of the flattened cornea at the first and second applanation (first and second A-length), corneal velocity during the first and second applanation moments (A1 V and A2 V), time from the start of the air pulse until the highest concavity of the cornea is reached, the central radius of curvature at the highest concavity, distance of the two surrounding ‘knees’ at the highest concavity as seen in the cross-sectional image, and maximum deformation amplitude at the corneal apex.

### 2.4 Statistical analysis

To obtain a sample size with an overall power of 80%, a two-tailed test or paired Student’s *t-*test was conducted. Based on the effect size of 0.5, we required 34 eyes (G*Power version 3.1.9.2, Franz Faul, Universität Kiel, Germany). The final study consisted of 57 participants (57 right eyes). IBM SPSS Statistics 20.0 (IBM Corp, United States) was used to perform the statistical analysis. The Kolmogorov–Smirnov test was applied to all data samples in order to check their normality. Data that followed a normal distribution were presented as mean ± SD; if not, data were presented as median (the lower quartile and upper quartile). Paired Student’s *t*-tests or Wilcoxon’s rank-sum tests were performed to determine significant differences in the biomechanical parameters. Bivariate correlations were evaluated using Pearson’s or Spearman’s correlation coefficients depending on the normality of the samples. Significantly correlated variables were introduced into a multiple linear regression, and the stepwise method was employed in the development of linear regression models. The statistical significance was accepted at *p* < 0.05.

## 3 Results

Twenty-nine males and twenty-eight females were included in the study, and their mean age was 22.7 ± 11.2 years.

Table 1 shows the biomechanical parameters of the subjects before and after eye rubbing. After 1 min of eye rubbing, SP-A1 decreased (*p* < 0.001). Higher deformation and deflection amplitudes (*p* < 0.001) and higher peak distances (*p* < 0.001) were detected. In addition, an earlier A1 time (*p* < 0.001), faster velocities (*p* < 0.001), and lower maximum inverse radius (*p* = 0.004) were also recorded. One week later, all the parameters had no statistical difference when compared with those before eye rubbing (Supplementary Table S1).

**TABLE 1:** Changes in biomechanical parameters after eye rubbing.

Biomechanical parameters	Before eye rubbing, mean ± SD [95% CI]	After eye rubbing, mean ± SD [95% CI]	*p*-value^a^
DA (mm)	1.04 ± 0.10 [1.01, 1.07]	1.09 ± 1.04 [0.82, 1.36]	<0.001
A1 T (ms)	7.50 ± 0.34 [7.41, 7.59]	7.34 ± 0.30 [7.26, 7.42]	<0.001
A1 V (m/s)	0.14 ± 0.02 [0.13, 0.15]	0.15 ± 0.02 [0.14, 0.16]	<0.001
PD (mm)	4.86 ± 0.29 [4.78, 4.94]	5.00 ± 0.27 [4.93, 5.07]	<0.001
HCDA (mm)	1.04 ± 0.10 [1.01, 1.07]	1.09 ± 0.10 [1.06, 1.12]	<0.001
A1DfA (mm)	0.09 ± 0.02 [0.08, 0.10]	0.10 ± 0.01 [0.10, 0.10]	0.033
HCDfA (mm)	0.86 ± 0.11 [0.83, 0.89]	0.90 ± 0.10 [0.87, 0.93]	0.012
Deflection amp. max. (ms)	0.90 ± 0.13 [0.87, 0.93]	0.93 ± 0.13 [0.90, 0.96]	0.045
Max. inverse radius (1/mm)	0.24 ± 0.13 [0.21, 0.27]	0.19 ± 0.06 [0.17, 0.21]	0.004
Pachy slope	55.10 ± 11.77 [52.04, 58.16]	53.12 ± 10.93 [50.28, 55.96]	0.046
bIOP (mmHg)	16.50 ± 2.55 [15.84, 17.16]	15.26 ± 1.97 [14.75, 15.77]	<0.001
SP-A1	123.53 ± 12.36 [120.32, 126.74]	112.79 ± 14.36 [109.06, 116.52]	<0.001

^a^ Paired Student’s *t*-tests.

IOP, intraocular pressure; DA, deformation amplitude; A1 T, time from the initiation of air puff until the first applanation; A1 V, corneal velocity during the first applanation; PD, peak distance; HCDA, maximum deformation amplitude; A1DfA, deflection amplitude of the first applanation; HCDfA, deflection amplitude of the highest concavity; deflection amp. max. (ms), time of the maximum deflection amplitude; bIOP, biomechanically corrected IOP; SP-A1, stiffness parameter at the first applanation.

An automatic linear modeling analysis of the effects of various factors, such as age, gender, diopter, IOP, and CCT, on the change of stiffness parameter at the first applanation (Δ SP-A1) after eye rubbing was performed. The results showed that two factors were associated with Δ SP-A1: myopic refractive state (B = −5.236, *p* = 0.010) and bIOP (*B* = 0.196, *p* = 0.016).

The demographic data of the patients are shown in Table 2 where myopic and emmetropic patients were analyzed separately and compared. The results of the analysis of the biomechanical parameters before and after eye rubbing in the emmetropia and myopia groups are shown in Tables 3, 4, respectively. In the emmetropia group, only IOP, bIOP, A1 T, A1 V, peak distance (PD), and the SP-A1 changed after eye rubbing (Table 3). In the myopia group, more parameters changed in response to eye rubbing. In all, the SP-A1 and corneal deformation parameters increased (DA, A1 V, PD, HCDA, HCDfA, deflection amplitude maximum, and A2 deflection area). The maximum inverse radius was the reciprocal of the maximum radius, therefore when it decreased after eye rubbing, it also meant that the cornea had become easier to deform by external force. Δ SP-A1 of the myopia group was 12.32% ± 7.19% larger than that of the emmetropia group 4.98% ± 7.63% (*p* < 0.001).

**TABLE 2:** Differences between emmetropia and myopia groups.

Parameter	Emmetropia group *n* = 29	Myopia group *n* = 28	*p*-value
Diopter	−0.20 ± 0.20	−3.56 ± 1.89	<0.001^a^
Age	26.8 ± 9.6	18.2 ± 11.4	0.003^a^
CCT	553.21 ± 29.92	533.75 ± 28.19	0.014^a^
Sex (percentage of females)	41.4%	53.6%	0.357^Δ^
Δ SP-A1	4.98% ± 7.63%	12.32% ± 7.19%	<0.001^a^

^a^ Independent samples *t*-test.

^Δ^Chi-square test.

^●^Mann–Whitney U test.

CCT, central corneal thickness. Δ SP-A1, percentage of changes of stiffness parameter at the first applanation after eye rubbing.

**TABLE 3:** Changes in biomechanical parameters after eye rubbing in emmetropia group.

Parameter	Before eye rubbing, mean ± SD [95% CI]	After eye rubbing, mean ± SD [95% CI]	*p*-value^a^
IOP (mmHg)	16.64 ± 3.00 [15.55, 17.73]	15.64 ± 2.22 [14.83, 16.45]	0.017
A1 T (ms)	7.51 ± 0.40 [7.36, 7.66]	7.39 ± 0.34 [7.27, 7.51]	0.021
A1 V (m/s)	0.14 ± 0.03 [0.13, 0.15]	0.15 ± 0.02 [0.14, 0.16]	<0.001
PD (mm)	4.82 ± 0.29 [4.71, 4.93]	4.93 ± 0.27 [4.83, 5.03]	0.01
Deflection amp. max. (mm)	0.88 ± 0.13 [0.83, 0.93]	0.92 ± 0.15 [0.87, 0.97]	0.006
bIOP	16.16 ± 3.17 [15.01, 17.31]	15.21 ± 2.22 [14.40, 16.02]	0.017
SP-A1	124.05 ± 13.97 [118.97, 129.13]	117.64 ± 14.72 [112.28, 123.00]	0.002

^a^ Paired Student’s *t*-tests.

IOP, intraocular pressure; A1 T, time from the initiation of air puff until the first applanation; A1 V, corneal velocity during the first applanation; PD, peak distance; deflection amp. max. (ms), time of the maximum deflection amplitude; bIOP, biomechanically corrected IOP; SP-A1, stiffness parameter at the first applanation.

**TABLE 4:** Changes in biomechanical parameters after eye rubbing in myopia group.

Parameter	Before eye rubbing, mean ± SD [95% CI]	After eye rubbing, mean ± SD [95% CI]	*p*-value^a^
IOP (mmHg)	16.68 ± 2.14 [15.89, 17.47]	14.89 ± 2.01 [14.15, 15.63]	<0.001
CCT (μm)	533.75 ± 28.19 [523.31, 544.19]	529.79 ± 29.51 [518.86, 540.72]	0.039
DA (mm)	1.05 ± 0.10 [1.01, 1.09]	1.11 ± 0.10 [1.07, 1.15]	<0.001
A1 T (ms)	7.49 ± 0.26 [7.39, 7.59]	7.29 ± 0.24 [7.20, 7.38]	<0.001
A1 V (m/s)	0.14 ± 0.02 [0.13, 0.15]	0.15 ± 0.01 [0.15, 0.15]	0.004
PD (mm)	4.90 ± 0.30 [4.79, 5.01]	5.05 ± 0.27 [4.95, 5.15]	0.002
HCDA (mm)	1.05 ± 0.10 [1.01, 1.09]	1.11 ± 0.10 [1.07, 1.15]	<0.001
HCDfA (mm)	0.86 ± 0.08 [0.83, 0.89]	0.93 ± 0.10 [0.89, 0.97]	<0.001
Deflection amp. max. (ms)	0.88 ± 0.13 [0.83, 0.93]	0.92 ± 0.15 [0.86, 0.98]	0.006
Max inverse radius (1/mm)	0.26 ± 0.15 [0.20, 0.32]	0.20 ± 0.07 [0.17, 0.23]	0.028
bIOP(mmHg)	16.85 ± 1.69 [16.22, 17.48]	15.31 ± 1.71 [14.68, 15.94]	<0.001
SP-A1	122.99 ± 10.67 [119.04, 126.94]	107.76 ± 12.31 [103.20, 112.32]	<0.001

^a^ Paired Student’s *t*-tests.

IOP, intraocular pressure; DA, deformation amplitude; A1 T, time from the initiation of air puff until the first applanation; A1 V, corneal velocity during the first applanation; PD, peak distance; HCDA, maximum deformation amplitude; HCDfA, deflection amplitude of the highest concavity; deflection amp. max. (ms), time of the maximum deflection amplitude; bIOP, biomechanically corrected IOP; SP-A1, stiffness parameter at the first applanation.

In the myopia group, the Δ SP-A1 was negatively correlated with age (r = −0.39, *p* = 0.04) and not correlated with the equivalent spherical diameter (r = 0.1, *p* = 0.613), CCT (r = −0.208, *p* = 0.289) or bIOP (r = 0.30, *p* = 0.121).

## 4 Discussion

According to the results, the cornea had become less stiff and easier to deform after rubbing. Similar results were achieved by Liu et al. (2011) by using ORA, who found that corneal hysteresis and corneal resistance in fact decreased after eye rubbing.

The eye rubbing–induced corneal biomechanical property changes may be explained as follows: first, eye rubbing may result in lamellar slippage of the cornea and instant reconstruction of the corneal collagen fibers (Dawson et al., 2008) may lead to changes in corneal biomechanical properties. Second, the corneal tissue exhibits viscoelastic behavior (Kallinikos and Efron, 2004; Chong and Dupps, 2021) and eye rubbing may result in agitation and reduced viscosity (softening) of the cornea (McMonnies, 2009). Third, by increasing the corneal temperature (Raizman et al., 2000), eye rubbing may reduce the bending resistance of the cornea (McMonnies, 2009). Besides, the CCT was the influencing factor for many corneal biomechanical parameters (Peña-García et al., 2016). McMonnies et al. (2010) found 18.4% reduction in epithelial thickness immediately after rubbing, and cell flattening, displacement of intercellular water from the rubbed area, the cytoplasm from ruptured cells, chains of wing cells, and mucin were suggested as possible thinning mechanisms. Changes in the CCT after eye rubbing may contribute to corneal biomechanical property changes.

In this study, when the participants underwent the Corvis ST test again 1 week later, all the parameters returned to the baseline level, which means that the cornea could recover completely after occasional eye rubbing. As to the recovery time frame, McMonnies et al. (2010) found that after eye rubbing, the CCT recovery to baseline thickness occurred between 15 and 30 min centrally and between 30 and 45 min mid-peripherally. The determination of the accurate recovery time of the corneal biomechanical properties requires further research. Previous studies have found that there were significant differences in corneal hysteresis (CH) between the emmetropia and myopia groups (Shen et al., 2008; Song et al., 2008; Bueno-Gimeno et al., 2014). Different CHs mean different times to return to their original shape after deformation (Broman et al., 2007). Lee et al. (2016) by using Corvis -ST found that there were differences in corneal biomechanical properties between the myopia and emmetropia groups. Therefore, we divided the patients into emmetropia and myopia groups to observe the different mechanical responses after eye rubbing.

The eye rubbing–induced biomechanical parameter changes in the myopia group differed from those observed in the emmetropia group. In the myopia group, there were more biomechanical parameter changes than in the emmetropia group. After eye rubbing, there was a greater decrease in corneal stiffness in the myopia group than in the emmetropia group. In ORA, low values of CH indicated a soft or floppy cornea, whereas in Corvis ST, smaller SP-A1 meant softer corneal stiffness (Roberts et al., 2016). Del Buey et al. (2014) found that CH was lower in the myopia groups than in the emmetropia groups, which means that the cornea is relatively softer in myopia. This might partly explain why the same eye rubbing action results in greater amplitude of SP-A1 deduction in the myopia group. The changes mean a more easily changed cornea in response to the external force. The changes in the CCT in our study were much smaller than that found in previous studies (McMonnies et al., 2010). McMonnies et al. (2010) found that the mean central thinning in response to 15 s of light to moderate rubbing was 11.6 μm. In our study, the changes in CCT in emmetropia were not statistically significant, and in myopia, the change was only 2.05 ± 8.73 μm. The difference might be attributed to the difference in the detection tool. In McMonnies et al. (2010) study, the Holden–Payor optical pachometer was used to measure the corneal thickness, while in our study, the Corvis ST was used. In the emmetropia group, CCT was unchanged after eye rubbing; there were still several biomechanical parameters that changed. Only CCT of myopic patients became thinner after eye rubbing, and it was the influencing factor for many corneal biomechanical parameters, such as the deformation amplitude and maximum inverse radius (Peña-García et al., 2016). Therefore, the difference in CCT change also partly explains why the responses of myopic and emmetropic patients to the eye rubbing differ.

The SP-A1 is a novel stiffness parameter, which conceptually describes resistance to corneal deformation (Liu et al., 2011). The stiffer the cornea, the more difficult for it to become deformed (Liu et al., 2011). In the myopia group, there was a negative correlation between age and ΔSP-A1, which means that the younger population was more easily influenced by eye rubbing. The different alteration amplitude of SP A1 in different age population may be related to differences in the corneal structure. A previous study (Daxer et al., 1998) had reported an increase in collagen fibril diameter, axial period, and intermolecular Bragg spacing with increasing age. These changes of the corneal structure along with age constrict the slippage of the corneal lamellae and consequently maintain the microstructure of the cornea. By contrast, a shorter collagen fibril diameter, axial period, and intermolecular Bragg spacing in the younger population may facilitate such slippage. The slippage caused by an external force such as eye rubbing may result in greater changes in corneal biomechanical properties.

According to (Gatinel, 2016; Gatinel, 2018) hypothesis, eye rubbing is an essential parameter in the pathophysiological mechanism that leads to keratoconus. Additionally, they noted that in patients with Marfan syndrome, the corneas are naturally thinner and less resistant, but they do not exhibit the central steepening that is seen in keratoconus. Instead, their corneas are flatter and exhibit more homogeneous tissue distension. This suggests that focal, repeated injury must account for the focal thinning and deformation observed in keratoconic corneas. Mechanical compression causes changes in cell morphology, reduces cell proliferation, triggers apoptosis, upregulates genes associated with extracellular matrix degradation, and downregulates corneal structural genes in human corneal fibroblasts. This research provides evidence that compressive stress has a significant impact on corneal keratocytes and suggests that this mechanical effect may be implicated in keratoconus development associated with chronic eye rubbing (Zhang et al., 2021). The prone sleeping position is a recently identified risk factor that may play a role in the laterality of the condition (Mazharian et al., 2020; de Azevedo Magalhães et al., 2021) that continuous pressure (head weight) can weaken the cornea and make it more vulnerable to eye rubbing.

During eye rubbing, when asked whether the force applied by the investigator was greater or smaller than what they apply in daily life, nearly all the participants answered that the force that they apply was greater than what was applied by the investigator. Therefore, daily eye rubbing may induce more dramatic changes than those observed in the present study. When asked about the frequency of their eye rubbing, all the participants reported that they only rub their eyes occasionally. When asked about their sleeping position, all of them reported that they habitually lay supine or lateral without eye oppression. Therefore, all participants’ baseline state could be deemed as intact.

Corneal thickness and biomechanical properties have an essential influence on the prediction of laser refractive surgery outcomes and evaluate the true IOP for glaucoma patients (Fernández et al., 2017). The Corvis ST has been widely used in clinics. From this study, the parameters achieved by the Corvis ST were easily influenced by eye rubbing and thereby made the measured results inaccurate. Taking into account that eye rubbing is a common physiological act in daily life, it is suggested that one should not rub their eyes before undergoing the Corvis ST examination.

In the present study, the corneas of myopic patients were vulnerable to rubbing-related changes, especially in the younger patients. The biomechanical properties of the cornea of younger myopic participants were affected more obviously by eye rubbing.

Opposite results have been reported in the Torres-Netto et al. (2022) study, where a custom-designed device was used to rub freshly enucleated porcine eyes with intact eyelids, applying a predetermined force 10 N on the eyelids, and repeating it 10,500 times with a frequency of 50 Hz in the vertical direction, which amounts to a time span of nearly 3 h. No significant corneal biomechanical changes were observed in the eyes subjected to repetitive and prolonged eye rubbing under *ex vivo* conditions when compared to the no-rub controls. There are several possible reasons that might lead to significantly different results. First, the *ex vivo* study was conducted using porcine corneas instead of human corneas, which are known to be thicker than human corneas. This difference in corneal thickness may underestimate the potential biomechanical effects of eye rubbing. Second, the distribution of forces during eye rubbing could also differ. In the normal orbital cavity, the counteracting force from adjacent soft tissues may be smaller. However, in an *ex vivo* model, a rigid wall with limited underlying tissues is present, which could potentially overestimate the biomechanical effects of eye rubbing. Third, the eye rubbing in our study was stroking closed eyes with pressure using the knuckles in a circular motion restricted to the cornea, which is deemed as the characteristic feature of eye rubbing in keratoconus, whereas in the Torres-Netto et al. (2022) study, the eye rubbing was carried out in the vertical direction. In addition, by increasing the corneal temperature (Raizman et al., 2000), eye rubbing may reduce the bending resistance of the cornea, and *ex vivo* rubbing could not influence the temperature.

Despite the absence of a control group, the study could rule out the possibility that the responses observed were simply a normal variation of the method. This can be attributed to several factors: first, the study had a large sample size, which enhanced the statistical power and reduced the likelihood of normal variations affecting the results. Second, the data collected 1 week after eye rubbing showed no significant difference when compared to the data collected before eye rubbing, indicating that the examination could distinguish changes caused by eye rubbing. Finally, there was a separate study that confirmed the reliable repeatability of the Corvis ST measurements (Wang X et al., 2021).

A limitation of this study is that we did not estimate accurately the time taken for the cornea to recover from the eye rubbing agitation and is therefore a problem when determining the superposition effect on the cornea from repeated eye rubbing agitations. Another limitation is that although only one investigator carried out the eye rubbing after training, the constancy of force still cannot be objectively measured or guaranteed.

We conclude that eye rubbing alters corneal biomechanical properties temporarily and softens the cornea, especially in myopic eyes of young individuals. We observed that all these changes returned to their initial levels after a week. The exact corneal biomechanical properties recovery time and effect of repeated eye rubbing on the cornea require further research.

## Data Availability

The original contributions presented in the study are included in the article/Supplementary Material; further inquiries can be directed to the corresponding authors.
