# Supplementary material for: Effect of eye rubbing on corneal biomechanical properties in myopia and emmetropia
Source: Front Bioeng Biotechnol. 2023 Jun 6;11:1168503. doi: 10.3389/fbioe.2023.1168503 (PMC10279860; doi:10.3389/fbioe.2023.1168503)
Supplement: Supplementary file 1 [file Table1.DOC]

**Supplementary table 1 The comparison of the biomechanical parameters before eye rubbing and one week later**

|  | Before eye rubbing  (mean±SD)  95% CI | One week after eye rubbing  (mean±SD)  95% CI | t | *P** |
| --- | --- | --- | --- | --- |
| IOP (mmHg) | 16.66 ± 2.59  [15.99, 17.33] | 16.78 ± 2.49  [16.13, 17.43] | -0.258 | 0.796 |
| CCT (um) | 543.65 ± 30.45  [535.74, 551.56] | 544.41 ± 28.28  [537.07, 551.75] | -0.138 | 0.890 |
| Def Amp Max (mm) | 1.04 ± 0.10  [1.01, 1.07] | 1.03 ± 0.10  [1.00, 1.06] | 0.742 | 0.459 |
| A1 Time (ms) | 7.50 ± 0.34  [7.41, 7.59] | 7.51 ± 0.31  [7.43, 7.59] | -0.043 | 0.965 |
| A1 Velocity (m/s) | 0.14 ± 0.02  [0.13, 0.15] | 0.13 ± 0.02  [0.12, 0.14] | 0.375 | 0.708 |
| A2 Time (ms) | 21.51 ± 1.14  [21.21, 21.81] | 21.44 ± 0.96  [21.19, 21.69] | 0.346 | 0.729 |
| A2 Velocity (m/s) | -0.25 ± 0.09  [-0.27, -0.23] | -0.25 ± 0.09  [-0.27, -0.23] | 0.193 | 0.846 |
| HC Time (ms) | 16.67 ± 0.68  [16.49, 16.85] | 16.69 ± 0.60  [16.53, 16.85] | -0.167 | 0.867 |
| Peak Dist (mm) | 4.86 ± 0.29  [4.78, 4.94] | 4.88 ± 0.30  [4.80, 4.96] | -0.434 | 0.664 |
| Radius (mm) | 6.48 ± 0.94  [6.24, 6.72] | 6.49 ± 0.82  [6.28, 6.70] | -0.059 | 0.953 |
| A1 Deformation Amp (mm) | 0.13 ± 0.01  [0.13, 0.13] | 0.12 ± 0.01  [0.12, 0.12] | 0.461 | 0.645 |
| HC Deformation Amp (mm) | 1.04 ± 0.10  [1.01, 1.07] | 1.03 ± 0.10  [1.00, 1.06] | 0.742 | 0.459 |
| A2 Deformation Amp (mm) | 0.42 ± 0.14  [0.38, 0.46] | 0.39 ± 0.14  [0.35, 0.43] | 0.905 | 0.366 |
| A1 Deflection Length (mm) | 2.12 ± 0.60  [1.96, 2.28] | 2.04 ± 0.67  [1.87, 2.21] | 0.615 | 0.539 |
| HC Deflection Length (mm) | 4.96 ± 2.03  [4.43, 5.49] | 5.20 ± 1.55  [4.80, 5.60] | -0.703 | 0.483 |
| A2 Deflection Length (mm) | 3.11 ± 1.74  [2.66, 3.56] | 3.53 ± 1.68  [3.09, 3.97] | -1.313 | 0.191 |
| A1 Deflection Amp (mm) | 0.09 ± 0.02  [0.08, 0.10] | 0.09 ± 0.01  [0.09, 0.09] | -0.926 | 0.355 |
| HC Deflection Amp (mm) | 0.86 ± 0.11  [0.83, 0.89] | 0.85 ± 0.11  [0.82, 0.88] | 0.102 | 0.918 |
| A2 Deflection Amp (mm) | 0.13 ± 0.11  [0.10, 0.16] | 0.13 ± 0.13  [0.10, 0.16] | -0.175 | 0.860 |
| Deflection Amp Max (mm) | 0.89 ± 0.13  [0.86, 0.92] | 0.88 ± 0.12  [0.85, 0.91] | 0.664 | 0.507 |
| Deflection Amp Max (ms) | 15.95 ± 2.16  [15.39, 16.51] | 16.47 ± 1.34  [16.12, 16.82] | -1.584 | 0.115 |
| Whole Eye Movement Max (mm) | 0.31 ± 0.15  [0.27, 0.35] | 0.28 ± 0.09  [0.26, 0.30] | 1.685 | 0.09 |
| Whole Eye Movement Max (ms) | 21.92 ± 1.38  [21.56, 22.28] | 21.77 ± 1.23  [21.45, 22.09] | 0.642 | 0.521 |
| A1 Deflection Area (mm^2^) | 0.17 ± 0.02  [0.16, 0.18] | 0.16 ± 0.02  [0.15, 0.17] | 1.458 | 0.147 |
| HC Deflection Area (mm^2^) | 2.97 ± 0.57  [2.82, 3.12] | 2.93 ± 0.70  [2.75, 3.11] | 0.362 | 0.717 |
| A2 Deflection Area (mm^2^) | 0.31 ± 0.41  [0.20, 0.42] | 0.33 ± 0.51  [0.20, 0.46] | -0.230 | 0.817 |
| A1 dArc Length (mm) | -0.01 ± 0.01  [-0.01, -0.01] | -0.01 ± 0.01  [-0.01, -0.01] | -0.176 | 0.860 |
| HC dArc Length (mm) | -0.09 ± 0.12  [-0.12, -0.06] | -0.06 ± 0.27  [-0.13, 0.01] | -0.775 | 0.439 |
| A2 dArc Length (mm) | -0.01 ± 0.05  [-0.02, 0.00] | -0.01 ± 0.03  [-0.02, 0.00] | 0.808 | 0.420 |
| dArcLengthMax (mm) | -0.15 ± 0.03  [-0.16, -0.14] | -0.14 ± 0.03  [-0.15, -0.13] | -1.531 | 0.128 |
| Max InverseRadius (1/mm) | 0.23 ± 0.12  [0.20, 0.26] | 0.23 ± 0.12  [0.20, 0.26] | 0.209 | 0.834 |
| DA Ratio Max (2mm) | 4.60 ± 1.55  [4.20, 5.00] | 4.56 ± 1.12  [4.27, 4.85] | 0.163 | 0.870 |
| PachySlope (um) | 55.10 ± 11.77  [52.04, 58.16] | 55.36 ± 11.89  [52.27, 58.45] | -0.123 | 0.901 |
| DA Ratio Max (1mm) | 1.64 ± 0.28  [1.57, 1.71] | 1.67 ± 0.29  [1.59, 1.75] | -0.453 | 0.651 |
| ARTh | 411.93 ± 106.99  [384.15, 439.71] | 403.34 ± 104.03  [376.33, 430.35] | 0.441 | 0.659 |
| bIOP | 16.49 ± 2.55  [15.83, 17.15] | 16.77 ± 2.39  [16.15, 17.39] | -0.597 | 0.551 |
| Integrated Radius (1/mm) | 8.60 ± 1.10  [8.31, 8.89] | 8.70 ± 1.27  [8.37, 9.03] | -0.496 | 0.620 |
| SP A1 | 123.52 ± 12.36  [120.31, 126.73] | 123.97 ± 13.30  [120.52, 127.42] | -0.190 | 0.848 |
| CBI | 0.10 ± 0.20  [0.05, 0.15] | 0.10 ± 0.17  [0.06, 0.14] | 0.245 | 0.806 |

*Paired student’s *t*-tests
